# Comment on “Urinary sodium-to-potassium ratio associates with hypertension and current disease activity in patients with rheumatoid arthritis: a cross-sectional study”: authors’ reply

**DOI:** 10.1186/s13075-021-02577-w

**Published:** 2021-07-21

**Authors:** Hiroto Minamino, Masao Katsushima, Yoshihito Fujita, Motomu Hashimoto

**Affiliations:** 1 Department of Diabetes, Endocrinology and Nutrition, Graduate School of Medicine, Kyoto University, 54 Shogoin, Kawahara-cho, Sakyo-ku, Kyoto-shi, Kyoto, 606-8507 Japan; 2 Japan Society for the Promotion of Science, 5-3-1 Kojimachi, Chiyoda-ku, Tokyo, 102-0083 Japan; 3 Department of Rheumatology and Clinical Immunology, Graduate School of Medicine, Kyoto University, 54 Shogoin, Kawahara-cho, Sakyo-ku, Kyoto-shi, Kyoto, 606-8507 Japan; 4 Department of Advanced Medicine for Rheumatic Diseases, Graduate School of Medicine, Kyoto University, 54 Shogoin, Kawahara-cho, Sakyo-ku, Kyoto-shi, Kyoto, 606-8507 Japan; 5 Department of Clinical Immunology, Graduate School of Medicine, Osaka City University, 1-4-3-13F Asahi-machi, Abeno-ku, Osaka-shi, Osaka, 545-8585 Japan

Dear editor,

Thank you for the comments on our previous article regarding the relationship between urinary sodium-to-potassium ratio (Na/K ratio), hypertension, and current disease activity in patients with rheumatoid arthritis (RA) [1]. Dr. Rastmanesh has raised some interesting points [2], and we appreciate the opportunity to respond to these comments.

The first comment was about the possibility of the presence of sodium-responsive subpopulation and the effect of potassium on hypertension. It is well-known that some hypertensive patients have a sodium-responsive mechanism and that potassium supplementation may improve hypertension in those patients. However, the most reliable method to diagnose sodium reactivity is the “salt intervention test,” which requires several days of intervention, and the intervention method and diagnostic criteria are not standardized [3]. Therefore, it is difficult to perform a sub-analysis in our study population. In our study, we aimed to determine whether dietary intake of sodium and potassium could affect the incidence of hypertension and disease activity in RA patients. To this end, we adopted the urinary Na/K ratio because it was more strongly correlated with blood pressure levels than either Na or K secretion alone in previous studies [4, 5]. We agree with Dr. Rastmanesh that it will be interesting to determine whether the effect of potassium on hypertension might be more pronounced in the sodium-sensitive subpopulation. It will also help optimize the therapy of hypertension in RA patients.

The second comment was the possibility that our findings might be influenced by the renin-angiotensin (RAA) system. Dr. Rastmanesh cited two papers, one stating that the RAA system influenced RA disease activity [6], and the other stating that renin was differentially expressed in RA and osteoarthritis (OA). However, the rationale from these papers is rather weak. The conclusion in the first paper was drawn based upon Spearman’s rank correlation coefficient (rho = 0.301, *p* = 0.034) for angiotensin II and DAS28 in one RA cohort (*N* = 50). While the second paper suggested the higher expression of renin in RA than OA synovium, another report found no difference in the serum renin and angiotensin-converting enzyme (ACE) levels among RA, OA, and healthy control [7]. Thus, the relationship between the RAA system and RA disease activity is not determined yet. Rather, recent advances in basic immunology discovered that serum Na concentration on its own could exacerbate inflammation by inducing inflammatory T helper cells (Th17) or inflammatory macrophages, which was included in the discussion section in our manuscript. Not only renin, but also various humoral hormones such as natriuretic peptides and adrenomedullin [8], could be involved in both inflammation and body fluid volume; thus, the effect of these humoral hormones on hypertension and RA disease activity may be worth studying in the future.

## Data Availability

Not applicable
